# Relevance and Comparability of Global Laboratory Molecular Detection of Influenza by the U.S. Department of War, 2025–2026

**Published:** 2026-09-20

**Authors:** Dara A. Russell, Marissa K. Hetrich, Kathleen E. Creppage, M. Shayne Gallaway

**Affiliations:** Global Emerging Infections Surveillance Branch, Armed Forces Health Surveillance Division, Public Health Directorate, Defense Health Agency, Silver Spring, MD: Ms. Russell, Ms. Hetrick, Dr. Creppage, CDR Gallway; Cherokee Nation Strategic Programs, Tulsa, OK: Ms. Russell, Ms. Hetrick, Dr. Creppage

## Abstract

The U.S. Department of War’s Global Emerging Infections Surveillance (GEIS) program conducts influenza surveillance within all geographic combatant commands to enhance U.S. force health protection and military readiness. This report compares laboratory-confirmed influenza data from the GEIS network with the World Health Organization (WHO) Global Influenza Surveillance and Response System (GISRS) from late 2025 through early 2026. Findings from the most recent 2025-2026 respiratory season show that while influenza trends were largely similar, with influenza A/H3N2 predominating, significant differences in percent positivity or subtype ratios emerged in Asia, East Africa, and South America. These discrepancies highlight the unique value of GEIS data, which fills critical surveillance gaps in strategic regions. This targeted surveillance provides crucial insights for mitigating infection risk to U.S. service members, informs annual vaccine strain selection, and ensures an early warning capability for emerging respiratory threats, directly supporting the readiness of the force.

Historically, respiratory pathogens have caused high morbidity rates for the U.S. military and, as a result, undermined military readiness. ^1^ Training and deployment environments are well suited for the spread of pathogens for several reasons, including the close proximity of personnel within these settings. ^2^ Monitoring influenza among service members and surrounding populations is crucial for force health protection due to its potential to cause severe illness and spread rapidly. To further support force health protection and maintain battle space awareness for the armed forces, the U.S. Department of War (DOW) supports influenza laboratory and epidemiological surveillance through military, local government, and academic partners at approximately 400 locations in over 40 countries.

**What are the new findings?**

During the 2025-2026 influenza season, subtype A/H3N2 was the most prevalent globally. While U.S. Department of War surveillance data largely concurred with the World Health Organization’s surveillance data, there were notable differences in positivity rates and subtype ratios identified by Department of War surveillance in Asia, East Africa, and South America that revealed unique regional trends.

**What is the impact on readiness and force health protection?**

Global influenza surveillance provides vital data for mitigating risk of infection in military service members. This surveillance informs not only effective vaccine development but targeted prevention as well, directly enhancing force health protection and ensuring military readiness through the prevention of influenza outbreaks.

Laboratory capabilities and personnel expertise are the foundations for effective responses to any infectious disease threat, and the DOW’s Global Emerging Infections Surveillance (GEIS) Branch of the Armed Forces Health Surveillance Division funds year-round surveillance, for both routine monitoring and possible outbreak investigations, of infectious diseases including influenza. The global network of DOW GEIS-supported laboratories generates critical data for force health protection and mitigation of infection risk to U.S. service members in all geographic Combatant Commands (CCMDs) **(Figure 1)** . GEIS laboratory surveillance extends extensive DOW epidemiological surveillance utilizing Military Health System (MHS) patient health care encounter data; and complements other existing global efforts. This continual surveillance occurs at both military hospitals and clinics (CONUS and OCONUS) and foreign military and civilian sites. Surveillance efforts prioritize active duty U.S. service members and high-risk groups such as recruits and deployed personnel, but other populations (e.g., beneficiaries, foreign military, foreign civilians) are also surveilled in priority locations to increase understanding and early warning for virulent strains associated with influenza-like-illness and severe acute respiratory infections, particularly in severe or hospitalized cases.

**FIGURE:**
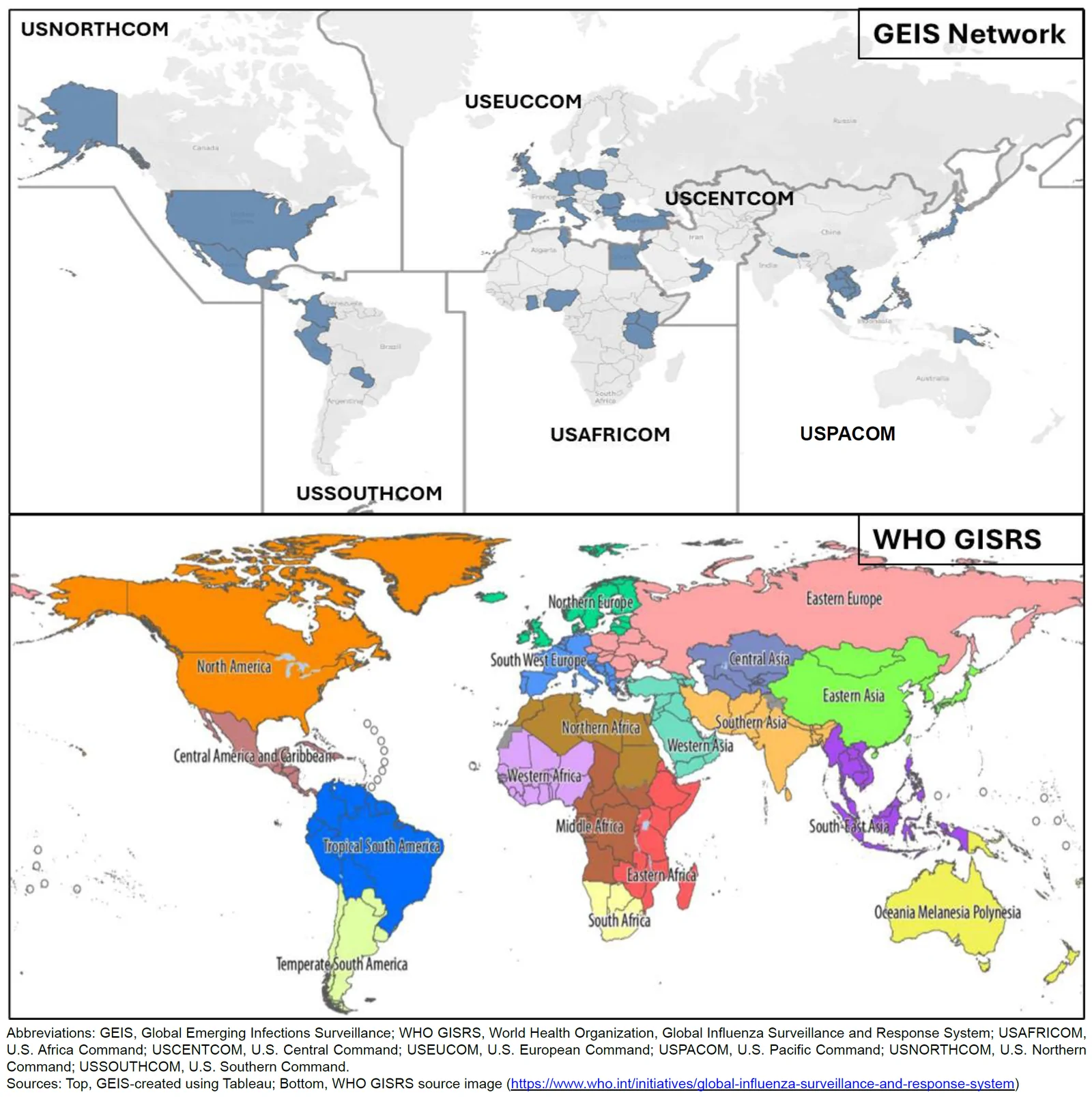
Geographic Coverage of Influenza Surveillance by DOW GEIS-funded Laboratory Network and WHO GISRS, 2025

This is achieved through extensive molecular detection and advanced genetic characterization capabilities to monitor how the influenza virus is circulating and potentially evolving. Across the GEIS Network (GEIS-N), molecular assays, including reverse transcription-polymerase chain reaction (RT-PCR), are used to identify influenza A and B and differentiate between influenza A subtypes (A/H1N1, A/H3N2). ^3^ The findings are shared with U.S. governmental interagency partners including the U.S. Centers for Disease Control and Prevention (CDC), in addition to the World Health Organization (WHO), with genomic data routinely uploaded to widely used public repositories including GenBank, GISAID, and the WHO Global Influenza Surveillance and Response System (GISRS). In many cases, these surveillance findings are uniquely informative: GEIS partner laboratories may be the only U.S. entity conducting infectious disease surveillance in regions where data would otherwise not exist, such as Djibouti.

Laboratory-confirmed influenza findings are disseminated routinely (immediately or monthly depending on risk threat) and are also aggregated and analyzed seasonally to contribute to greater scientific decision-making for seasonal influenza strain selection for the Northern Hemisphere influenza vaccine. Prior to the Vaccines and Related Biological Products Advisory Committee (VRBPAC) ^4^ meetings for the Northern Hemisphere and U.S influenza strain recommendations, the GEIS Program Office conducts a comparative analysis of circulating influenza subtype ratios between DOW GEIS partner laboratories and WHO GISRS. ^5^ This study assessed regional differences by analyzing and comparing laboratory confirmed influenza subtypes from the most recent 2025-2026 respiratory season between GEIS-N and WHO GISRS.

## Methods

### Data sources

The GEIS Program Office developed a procedure and initiated monthly standardized RT-PCR respiratory surveillance data collection in early 2020, anticipating a need for readily available information in response to the emerging COVID-19 pandemic. A standardized data collection form was developed to capture respiratory pathogens represented on widely used RT-PCR panels (updated again prior to the 2024-2025 respiratory season). As of 2026, RT-PCR respiratory testing results (including influenza by subtype) are collected from 10 GEIS-funded partner laboratories executing 30 different surveillance projects of varying scopes in 37 countries across all CCMDs. These include U.S. Northern Command (NORTHCOM), at the Department of Defense Global Respiratory Pathogen Surveillance Program and Naval Health Research Center; U.S. Southern Command (SOUTHCOM), at the Naval Medical Research Unit (NAMRU) South; U.S. European Command (EUCOM), at Landstuhl Regional Medical Center, Germany and Walter Reed Army Institute of Research (WRAIR) Europe Middle East; U.S. Africa Command (AFRICOM), at WRAIR Africa and NAMRU EURAFCENT, Ghana; U.S. Central Command (CENTCOM), at NAMRU EURAFCENT, Cairo, Egypt; and U.S. Pacific Command (PACOM), at NAMRU Indo-Pacific, WRAIR Armed Forces Research Institute of Medical Sciences (AFRIMS), and Public Health Command Pacific at Camp Zama, Japan; Tripler Army Medical Center, Honolulu, Hawai’i, and Joint Base Lewis McChord, Tacoma, Washington.

Routine reporting results are aggregated by CCMD to assess the frequency and distribution of viral subtypes over time. These routine respiratory surveillance data are used to: 1) develop or inform routine products for GEIS audience members, 2) inform *ad hoc* inquiries from CCMDs and other key stakeholders, and 3) provide context for the operational environment as needed, whether during times of conflict, military engagement, or public health emergency.

Among 135 WHO member states, WHO GISRS ^5 , 6^ serves as a global network that monitors influenza transmission and facilitates data sharing. Surveillance data generated through WHO GISRS include FluNet, ^7^ FluID, ^8^ and RespiMart. ^9^ FluNet is a global web-based data platform established in 1997 that is updated weekly to compile laboratory-confirmed influenza surveillance data ^10^ ; the GEIS Program Office downloads these data once annually for comparison with GEIS laboratory-confirmed influenza surveillance data across CCMDs.

WHO transmission zones and CCMD areas of responsibility (AORs) serve different purposes and, as a result, do not mirror each other. WHO transmission zones were established to coordinate international public health efforts and disease surveillance, whereas CCMDs are U.S. Armed Forces-defined regions designed to organize and implement military operations and security cooperation activities.

As a DOW combat support agency, for this analysis GEIS realigned the WHO transmission zones to match DOW CCMD AORs to ensure maximum applicability for military service members. Some WHO regions were combined into broader groupings: 1) data from Northern Europe, Eastern Europe, South West Europe, and Western Asia (not including Israel) were combined to form ‘Europe’, 2) data from Central America and the Caribbean, Temperate South America, and Tropical South America were combined to form ‘South America’, 3) data from Northern Africa, Western Asia (Israel), Southern Asia (Afghanistan, Iran) were combined to form ‘Middle East’, 4) data from Eastern Asia, Southern Asia (not including Afghanistan, Iran), and South East Asia were combined to form ‘Asia’, while 5) data from North America, East Africa, and West Africa were unchanged and remained aligned to their regions.

### Analysis

Epidemic curves or epicurves (i.e., a histogram or bar chart) were created to display the distribution of positive specimens over time associated with influenza. Time intervals are displayed on the x-axis (horizontal axis) with specimen counts displayed on the y-axis (vertical axis). These provide information on the counts, patterns of spread, time trends, and periods of increased positivity.

Influenza RT-PCR data generated from GEIS-funded surveillance activities were standardized to WHO regions by country of sample collection. Counts of influenza B Victoria and B/not-subtyped specimens were summed to create a single measure of influenza B positives for comparison to the equivalent influenza B sum in the WHO GISRS dataset. Counts for total specimens tested, specimens positive for influenza A subtypes (H1N1, H3N2, A/not-subtyped), all influenza B, and A/B co-infections were summed by WHO region and epi week. ^11^ Influenza percent positivity was calculated for each WHO region and epidemiological week by summing the number of H1N1, H3N2, A/not-subtyped, all influenza B, and A/B co-infection positive specimens divided by the total number of specimens tested. Co-infections were included in percent positive calculations (but not graphically represented) because co-infections were not separately defined in the WHO GISRS dataset.

WHO GISRS is based primarily on hemagglutinin (HI) RT-PCR, thus data counts for influenza A ‘H1’ and ‘H1N12009’ were combined into a single influenza A/H1N1 measure. WHO influenza A ‘H3’ was considered the equivalent of GEIS influenza A/H3N2. Similar to the GEIS calculations, counts for total specimens tested, specimens positive for influenza A subtypes, and influenza B were summed by WHO region and epidemiological week; and influenza percent positivity was calculated for each WHO region and epidemiological week by summing the number of H1N1, H3N2, A/not-subtyped, and influenza B positive specimens divided by the total number of specimens tested. The date of week start was converted to the date of the week end by adding 5 days to harmonize date ranges across GEIS and WHO data for data plotting.

To establish baseline seasonal patterns and directly compare the most recent respiratory seasonal findings, data from the last 2 respiratory seasons and the most current respiratory seasons were included in the analysis (weeks ending October 7, 2023 through January 31, 2026). Counts of positive specimens by epidemiological week were graphed for each WHO region and percent positivity calculations were overlaid for each corresponding timepoint. Weighted Dynamic Time Warping (wDTW) ^12 , 13^ analysis was then used to assess the similarity between the 2 trends of percent positivity (GEIS and WHO GISRS) for the most current respiratory season (September 2025–January 2026). A non-parametric permutation test was employed to allow for the statistical calculation of a *p* -value indicating the likelihood that the distance between temporal trends occurred by random chance. Given the low statistical power, a *p* -value of less than 0.20 was used as a threshold for significance. Data were processed and graphed using R Statistical Software (version 4.5.1). ^14^

## Results

Laboratory-confirmed influenza percent positivity temporal trends from late 2023 through early 2026 were generally similar between GEIS-N and WHO GISRS **(Figure 2)** . Prior GEIS-N and WHO GISRS respiratory season data (2023-2025) provide context for baseline observations and evolving trends and subtypes. Overall percent positivity ranged 0–38% for GEIS-N and 0–25% for WHO GISRS for all regions combined. The highest percent positivity consistently reported by GEIS was in Asia.

**FIGURE:**
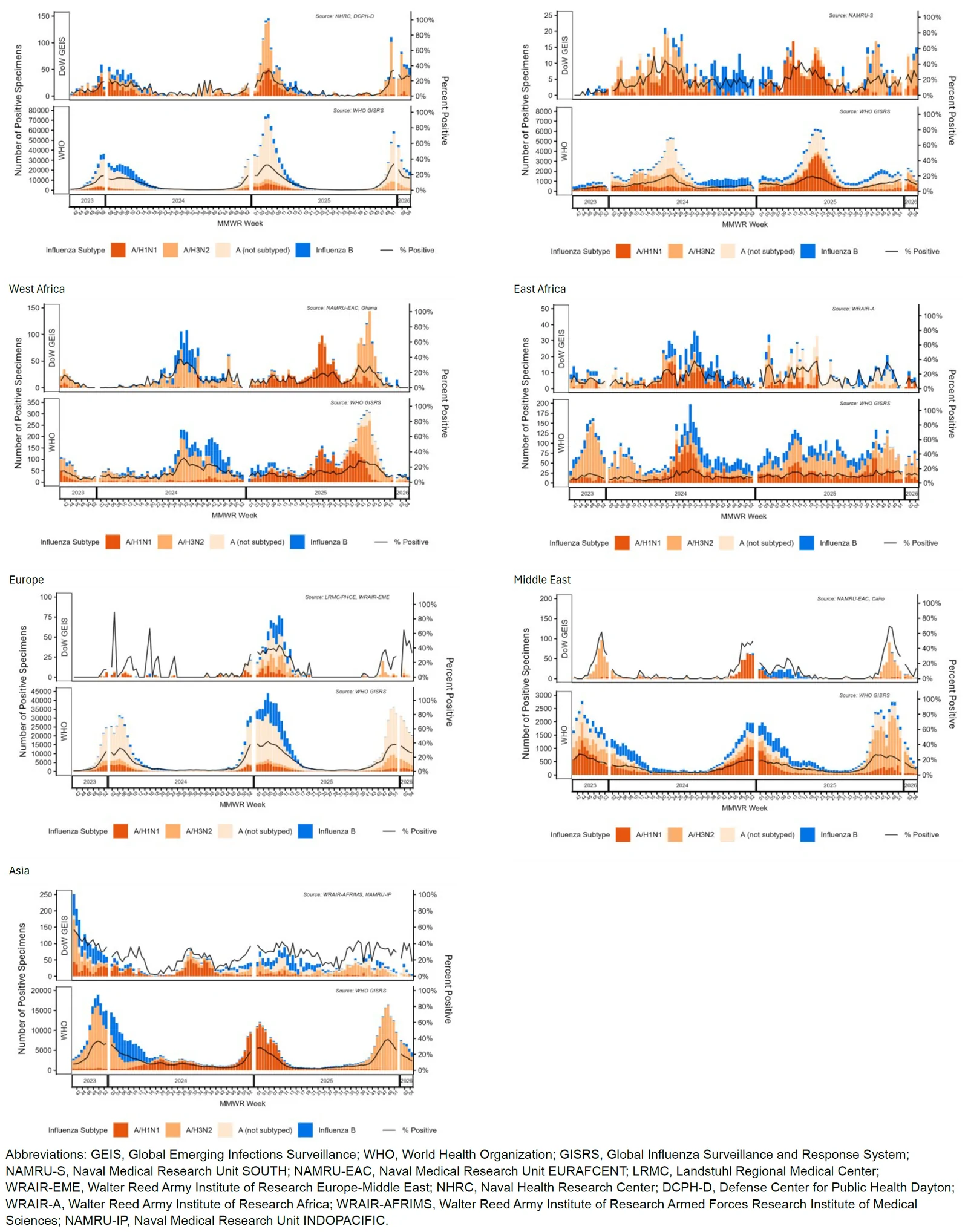
Influenza Specimen Detection Numbers and Percent Positivity by GEIS-funded Laboratories and WHO GISRS, October 14, 2023–February 7, 2026

During the most recent respiratory season (2025-2026, **Table 1** ), the percent positivity observed between GEIS-N and WHO GISRS regions was statistically different in North America ( *p* <0.01), Europe ( *p* <0.01), West Africa ( *p* <0.01), and the Middle East ( *p* <0.05), and statistically similar in South America ( *p* =0.33), East Africa ( *p* =0.95), and Asia ( *p* =0.99). Notable differences in subtype-specific percent positivity were detected between GEIS-N and WHO GISRS regions: GEIS reported a higher percent positivity than WHO for influenza A/H3N2 in Europe (13.3% vs. 4.4%), South America (11.9% vs. 3.6%), and North America (10.4% vs. 2.5%), whereas WHO reported a higher percent positivity than GEIS for influenza A/H3N2 in Asia (20.0% vs. 11.3%); in East Africa and Asia, GEIS detected a higher percent positivity than WHO for influenza B.

**TABLE 1.** Influenza Percent Positivity by Geographic Region of GEIS-funded Laboratory Specimens and WHO GISRS, September 28, 2025–February 7, 2026

Geographic Region	GEIS ^a^	GISRS	*p* -value ^b^
North America	13.6	15.5	<0.01
A/not-subtyped	0.0	11.7	
A/H1N1	0.9	0.4	
A/H3N2	10.4	2.5	
B	1.1	0.9	
South America	19.4	10.4	0.33
A/not-subtyped	0.4	3.6	
A/H1N1	1.7	1.7	
A/H3N2	11.9	3.6	
B	1.6	1.5	
West Africa	16.1	19.2	<0.01
A/not-subtyped	0.2	3.6	
A/H1N1	1.8	3.8	
A/H3N2	13.2	11.0	
B	0.8	0.8	
East Africa	17.0	13.1	0.95
A/not-subtyped	7.3	0.8	
A/H1N1	1.7	4.1	
A/H3N2	2.9	7.0	
B	5.1	1.3	
Europe	18.2	24.3	<0.01
A/not-subtyped	2.9	18.5	
A/H1N1	1.7	1.3	
A/H3N2	13.3	4.4	
B	0.0	0.2	
Middle East	29.3	20.5	<0.05
A/not-subtyped	0.0	4.0	
A/H1N1	1.3	2.0	
A/H3N2	17.3	13.3	
B	0.5	1.2	
Asia	30.4	21.2	0.99
A/not-subtyped	10.4	0.1	
A/H1N1	1.1	0.3	
A/H3N2	11.3	20.0	
B	7.3	0.7	

Abbreviations: GEIS, Global Emerging Infections Surveillance; WHO, World Health Organization; GISRS, Global Influenza Surveillance and Response System; DOW, Department of War.

a Influenza A, B co-infections included in calculation of overall percent positivity by region for DOW GEIS surveillance.

b *p* -value corresponding to trend comparisons using dynamic time warping analysis

Subtype ratios varied some between the GEIS-N and WHO GISRS from late 2023 through early 2026, with a greater ratio of WHO specimens being classified as influenza A (A/not-subtyped), likely a facet of laboratory testing processes by GEIS partner laboratories contributing to some WHO regions (e.g., West Africa, North America, South America, Europe, Middle East). During the most recent respiratory season (2025-2026), the subtype ratios observed between GEIS and WHO regions were generally similar in Asia, East Africa, Europe, North America, and South America, while dissimilar in the Middle East and West Africa. In the Middle East there are notable differences in the ratio of influenza A/H1N1 (33% and 21%) and A / not-subtyped (33% and 43%) specimens detected by GEIS and WHO, respectively, while influenza A/H3N2 accounts for about one third of all GEIS (33%) and WHO (36%) specimens in both systems. In West Africa, there are also notable differences in the ratio of influenza A/H1N1 (10% and 22%) and A / not-subtyped (59% and 43%) specimens detected by GEIS and WHO, respectively.

## Discussion

Data collected from the GEIS-N are similar to what is seen in WHO GISRS during the most recent respiratory season (October 2025–January 2026). In some cases, GEIS-PL surveillance contributions are included in submissions by GEIS laboratories are closely aligned/partnered with (e.g., Ghana, Malaysia, Uganda, Vietnam) and in at least 1 instance make up a large proportion of the WHO GISRS data (NAMRU EURAFCENT Ghana influenza surveillance executed alongside CDC surveillance efforts). Differences between DOW GEIS surveillance and WHO GISRS highlight the importance of using the GEIS-N to identify and prioritize key samples for sequencing and advanced characterization. GEIS-N surveillance captures influenza circulating in over 30 countries, including several locations that border other countries, to maximize influenza specimens of viral genetic diversity. GEIS-N also includes a large service member population that is highly vaccinated (for influenza), ^15^ healthy, and has broad access to health care.

During each global influenza season approximately 1 billion cases occur, resulting in 3–5 million cases of severe illness and 290,000–500,000 deaths. ^16^ During the most recent season (October 2025–January 2026), percent positivity varied by geographic region, with highest GEIS-N-reported positivity in Asia and the Middle East and highest WHO GISRS-reported positivity in Asia and Europe. ^17^ Overall, in both systems, influenza A viruses predominated, with low levels of influenza B detected. Among influenza A viruses with a confirmed subtype, influenza A/H3N2 was the most prevalent in most regions.

The RT-PCR findings from GEIS-N are further enhanced and contextualized by GEIS-funded next-generation sequencing and bioinformatics (within an evolving and modernized data infrastructure). These program initiatives are a set of linked activities that support and enhance the effects and relevance of DOW influenza surveillance. Routine surveillance is important for establishing baseline trends so anomalies can be detected more rapidly. GEIS partner laboratories (with critical infectious disease surveillance capabilities) are strategically positioned in countries and regions vital to DOW operations for early, accurate detection of emerging infections. GEIS-N can also be leveraged to detect and characterize novel influenza or other emerging respiratory infections; for example, the next generation sequencing and bioinformatics capabilities that already existed were scaled up to monitor SARS-CoV-2 variants during the COVID-19 pandemic as the virus evolved and spread. ^18^

Maintaining awareness of influenza activity and reinforcing preventive measures aligned with existing respiratory illness protocols are crucial for disease control. U.S. service member and beneficiary populations are the GEIS-N target populations of interest, and these findings aid in establishing a clear link to force health protection to ultimately improve health through the prevention, mitigation, and control of influenza virus transmission. Compliance with immunization requirements should be monitored to prevent transmission of influenza and other respiratory pathogens. ^19^

These findings should be considered alongside the following limitations. Both GEIS and WHO GISRS provide broad overviews across global regions, however the differences highlight the importance of understanding local laboratory surveillance practices. While the majority of GEIS partner laboratories currently use a respiratory viral panel (or a panel in combination with single pathogen testing), they are not formally required to test for all respiratory patho gens. ^3^ Likewise, sample collection and RT-PCR testing varies across GEIS surveillance projects (e.g., site collection and transport, reagent kits, shipping necessity), whereas WHO GISRS collects samples in transport media that allows specimens to be shipped to reference laboratories where they can be used for reagent production, virologic characterization, vaccine components, and other uses.

These results reflect surveil lance data only reported directly to the GEIS program office by funded GEIS partner laboratories, thus it likely is an under-estimate of the true incidence and may not be representative of all respiratory infections among all DOW active component personnel globally or across the MHS. They are, however, indicative of subtypes circulating in the specified geographic populations and surveillance sites.

The GEIS and WHO GISRS target population characteristics and geographies are notably different, and we were unable to adjust for all possible confounders in this analysis that may have influenced the results. Regrouping of WHO GISRS transmission zone surveillance data into DOW CCMD AORs rather than using the established WHO GISRS transmission zones (representing well-defined seasonality patterns) may have obscured some localized transmission patterns and limit direct comparability of the 2 surveillance systems. In future analyses, we will explore how regrouping GEIS data to match WHO GISRS transmission zones will change the observed results. Finally, the DOW does not conduct influenza surveillance in every WHO member country, thus some variability is expected across regions depending on the degree of overlap between CCMDs and WHO influenza transmission zones.

Closely monitoring influenza infections can inform vaccine decision-making with broad global implications. DOW GEIS surveillance findings are unique because they include laboratory-confirmed data on a variety of populations in locations where there may be gaps in U.S. data. GEIS-N also contributes to broader public health security objectives by establishing enduring, reliable partnerships with nations that have a shared stake in the security and prosperity of each region and facilitate broader DOW and U.S. Government collaborations or host nation surveillance. Routine public health surveillance is critical for preparedness against emerging influenza viruses.
